# Shared Decision Making in Immigrant Patients

**DOI:** 10.7759/cureus.1461

**Published:** 2017-07-12

**Authors:** Claudia C Dobler, Gabriela Spencer-Bonilla, Michael R Gionfriddo, Juan Pablo Brito

**Affiliations:** 1 Knowledge and Evaluation Research Unit, Mayo Clinic, Rochester, Minnesota; 2 Center for Pharmacy Innovation and Outcomes, Geisinger Health System, Forty Fort, Pennsylvania

**Keywords:** shared decision making, communication, immigrants, foreign language

## Abstract

Communication is at the core of shared decision making, and communication difficulties are therefore barriers to using shared decision making in clinical practice. In clinical encounters with immigrant patients from culturally and linguistically diverse backgrounds, a number of communication challenges arise, which can be obstacles to the implementation of shared decision making. Here, we discuss some of these challenges, possible solutions and research required to address identified knowledge gaps.

## Editorial

Shared decision making (SDM), the work that patients and physicians do together in conversation to arrive at the best possible solution to address the patient’s health care situation, has been widely advocated as an important component of patient-centered care. Translating this ideal into reality has proven challenging [1]. Several potential barriers to implementation of SDM in clinical practice have been identified, such as clinicians’ (questionable) preconceptions that SDM takes too much time, that SDM is not compatible with clinical practice guidelines or that in SDM patients are left to make decisions alone [2]. A number of specific challenges arise in the context of intercultural and interlinguistic SDM, which may be particularly pertinent to immigrant populations. Some of the challenges of SDM in an intercultural context have been summarized in a paper by Suurmond and Seeleman [3]. These challenges include 1) language barriers, need for interpreters, 2) differences in health beliefs and concepts of illness between the patient and clinician, 3) differences in role expectations, e.g. an apparent preference for a paternalistic approach or desire for family-centered model of decision making, 4) consultation situation (e.g., time constraint and lack of culturally adapted patient information), and 5) low health literacy. Here, we take a closer look at some of these challenges, which need to be addressed when developing SDM tools for immigrant patients, e.g. discussion of latent tuberculosis treatment.

A core component of SDM is communication. When clinicians and patients have to communicate through an interpreter, the work of SDM is complicated by: incorporating a third party into a sometimes intimate conversation, disruption of a typical communication flow, lengthening of the medical encounter, and the telephone effect when interpreters engage in interpretation and curation of language rather than pure translation. Interpreters, whether professional or lay, may make judgments about which information is important to convey to patients (and back to the clinician) and which information is not. Little is known about how this form of triadic communication affects the process of SDM and the extent to which interpreters’ knowledge, attitudes, and beliefs affect SDM and the use of SDM tools in clinical encounters. A recent study examining the use of interpreters and SDM for osteoarthritis found that treatment discussions mainly occurred between the clinician and the interpreter [4]. Patients had only minimal participation in the discussion with an average of four words articulated when they had an opportunity to speak, indicating that patients did not have a significant role in discussing treatment options.

In addition to differences in language, patients from different cultural backgrounds may have illness narratives and values and preferences which do not align with those of their clinicians. Concepts about the meaning and causes of illness and how it should be treated may differ. Patients might trust traditional healers more than Western medicine. Clinicians’ understanding of the ‘sick role’ with its rights (e.g., temporary relief from usual workload) and obligations (e.g., follow doctors’ orders) may not be consistent with that of their patients. Providing care is also complicated by the fact that immigrants, especially those newly arrived in the destination country and with limited socio-economic resources, can have pressing material needs and concerns like providing for the daily needs of their families. A holistic approach to improving health and well-being must also take into account each patient’s context in the decision-making process further emphasizing the importance of SDM in this situation.

A single solution will not address all of these barriers, and more research is needed to determine the effectiveness of available interventions. For conversations that require interpreters, more research is needed around the dynamics of these triadic conversations as well as strategies for facilitating SDM in this context. Research is also required to find models of SDM that do not only facilitate collaborative deliberation between two individuals (the patient and the clinician), but facilitate the inclusion of family members and caregivers into the decision making process. To adapt to cultural differences, group education classes (in which patients from similar cultural backgrounds learn about basic medical principles pertinent to their health concern) or shared visits in addition to individual encounters may help create a cohesive narrative between patients and clinicians. This strategy is currently being implemented by one of our collaborators in China. As many cultures have a family-centered model of decision making, patients’ families could be integrated into these group classes as well. Clinicians need to be provided with training opportunities for SDM including SDM with patients from different cultural backgrounds. Interpreters should be made aware of the goals and mechanisms of SDM, so that they can provide interpretation service in line with these.

At times, SDM conversations will need to incorporate existential or practical needs that extend beyond a specific medical decision and may limit patients’ capacity to engage in SDM. Thus, components of the ICAN (Instrument for Patient Capacity Assessment) tool, a discussion aid for clinicians to use together in visits with their patients to better understand their patients’ context (including their socioeconomic status and literacy) and situation including goals, priorities, capacity, and burden [5], may be a useful addition to a SDM intervention in this disease context.

While ongoing refugee crises throughout the world have highlighted the limitations of current approaches to SDM, these challenges exist to varying degrees in all encounters; we all have our own microcultures and idiosyncrasies. Discovering how to communicate with one another in an effective, respectful, compassionate, and empathic manner is essential for the realization of the promises of patient-centered care.

This is a modified version of a blog post, first published on: http://shareddecisions.mayoclinic.org/blog/
